# *Trichosanthes cucumerina* Linn. improves glucose tolerance and tissue glycogen in non insulin dependent diabetes mellitus induced rats

**DOI:** 10.4103/0253-7613.42301

**Published:** 2008-06

**Authors:** H. Kirana, B.P. Srinivasan

**Affiliations:** Delhi Institute of Pharmaceutical Sciences and Research (DIPSAR), MB Road, Pushp Vihar, Sector-III, New Delhi-110 017, India

**Keywords:** Glycogen, Non Insulin Dependent Diabetes Mellitus, oral glucose tolerance test, *Trichosanthes cucumerina*

## Abstract

**Objective:**

To study the effect of *Trichosanthes cucumerina* Linn. on non insulin dependent diabetes mellitus induced rats.

**Materials and Methods:**

Non Insulin Dependent Diabetes Mellitus (NIDDM) was induced by administering streptozotocin (90 mg/kg, i.p.) in neonatal rat model. NIDDM animals were treated with aqueous extract of *Trichosanthes cucumerina* (100 mg/kg/day) orally for six weeks. Parameters such as fasting blood glucose, Oral Glucose Tolerance Test (OGTT) and tissue glycogen content were evaluated.

**Results:**

Aqueous extract of *Trichosanthes cucumerina* significantly (*P*<0.01) decreased the elevated blood glucose of NIDDM induced rats. OGTT of NIDDM animals showed glucose intolerance. Blood glucose of diabetic animals reached peak at 45 min and remains high even after 2h. In case of *Trichosanthes cucumerina* treated group, the blood glucose reached peak level at 30 min, followed by decrease in glucose level up to 2h. The drug has significantly (*P*<0.01) reduced the postprandial blood glucose of diabetic animals. Glycogen content of insulin dependent tissues such as liver and skeletal muscle was found to be improved by 62% and 58.8% respectively with *Trichosanthes cucumerina* as compared to NIDDM control.

**Conclusion:**

Studies revealed that, *Trichosanthes cucumerina* possess antidiabetic activity. The drug improved the oral glucose tolerance of NIDDM subjects. Increase in tissue glycogen content indicates the effect of the drug on the uptake of glucose by the peripheral tissues to reduce insulin resistance of NIDDM.

## Introduction

Non Insulin Dependent Diabetes Mellitus (NIDDM) also called as type 2 diabetes is a complex metabolic disorder that involves abnormalities in both insulin secretion and action at peripheral tissues.[1] It is a more prevalent form of diabetes and responsible for 90% of the disease. In NIDDM, the kinetics of insulin release in response to meal or glucose is altered. So, postprandial blood glucose remains high and leads to glucose intolerance. Postprandial hyperglycemia plays an important role in the development of diabetic complications.[2] Poor glycogen content in insulin dependent tissues such as liver, skeletal muscle and adipose tissues were observed in NIDDM due to insulin resistance.[3]

*Trichosanthes cucumerina* Linn. belonging to family Cucurbitaceae is an annual climber and widely distributed in southern parts of India. Traditionally, decoction of the stem, leaves and aerial parts were used in the treatment of diabetes and inflammatory diseases.[4] The major active constituents of the drug are triterpenoid saponins viz., cucurbitacins.[5] On the above evidence, the present investigation was planned to study the effect of aqueous extract of *Trichosanthes cucumerina* Linn. on NIDDM induced rats.

## Materials and Methods

### Collection and authentication of plant material

*Trichosanthes cucumerina* was collected from local market of Udupi (Karnataka) during the month of November-December. Plant material was dried under shade at temperature less than 40°C. The drug was authenticated by botanist at the National Institute of Science Communication and Information Resources (NISCAIR), New Delhi. Specimen of the drug was deposited (Accession No. 5065) at Herbarium and Museum, NISCAIR, New Delhi.

### Preparation of aqueous extract[4–5]

The dried plant material was grounded into a moderately coarse powder using domestic electric grinder. One part of the powdered drug was boiled with sixteen parts of water for a period of 15 min. It was filtered hot through muslin cloth. The filtrate was evaporated under reduced pressure and dried to obtain 6.8% of aqueous extract.

### Animals

*Wistar* albino rats (140-160 g) and *Swiss* albino mice (20-22 g) of either sex were housed under standard laboratory conditions of light and dark cycles of 7.00 am to 7.00 pm, temperature of 25 ± 2°C and 55% relative humidity. The animals were given standard rat pellet and tap water *ad libitum*. The study protocol was approved by Institutional Animal Ethical Committee, DIPSAR, New Delhi, India.

### Acute toxicity study

*Swiss* albino mice (20-22 g) of either sex were divided into five groups of six animals each. Animals were fasted overnight but allowed free access to water prior to the experiment. Aqueous extract of *Trichosanthes cucumerina* at different dose levels, i.e., 0.5, 1.0, 1.5, and 2.0 g/kg body weight was administered once orally to respective experimental groups. The volume of each administered dose did not exceed 1 ml. Control group received 1 ml of distilled water. The mice were then observed for 24h and mortality was recorded. Median lethal dose (LD_50_) was determined according to Karber's method.[6]

### Streptozotocin induced neonatal rat model for NIDDM[7]

NIDDM was induced by administering streptozotocin (90 mg/kg i.p.) in two-day-old neonatal rats. After six weeks of streptozotocin injection, animals showing the fasting blood glucose level more than 140 mg/dl were considered as NIDDM positive.[8]

### Experimental groups

*Wistar* albino rats of either sex were randomly allotted into four groups of six animals (n=6) each. Equal number of males and females were maintained in each group and caged separately. Group I served as normal and received distilled water. Group II served as NIDDM control and received distilled water. Group III was NIDDM animals treated with aqueous extract of *Trichosanthes cucumerina* at a dose of 100 mg/kg/day by oral route. Group IV was NIDDM animals treated with glibenclamide at a dose of 1.5 mg/kg/day by oral route. The drug treatment was carried out on every day morning with the help of oral catheter for a period of six weeks.[3,5] Fasting blood glucose and body weight were determined after 1 week, 2 weeks, 4 weeks and 6 weeks of drug treatment. After six weeks of drug treatment, Oral Glucose Tolerance Test (OGTT) and glycogen content in tissues such as liver and skeletal muscle were evaluated.

### Estimation of blood glucose[9]

Blood samples were withdrawn from overnight fasted animals and centrifuged at 3000 rpm for 10 min, at 4°C in cooling centrifuge (Remi, C-24 BL, Mumbai, India). Glucose in serum was estimated by Glucose Oxidase and Peroxidase (GOD-POD kit) method. The intensity of red quinoneimine was measured at 540 nm in Autoanalyzer (Logotech, Tecno 168, Italy).

### Oral glucose tolerance test (OGTT)[10]

Overnight fasted animals were challenged by loading glucose solution orally at a dose of 2.5 g/kg body weight. Blood samples were withdrawn immediately after glucose load (0 min reading) and at 15, 30, 45, 60, 75, 90 and 120 min of oral glucose load. Blood glucose was estimated by Glucose Oxidase and Peroxidase (GOD-POD kit) method. Glucose tolerance curves were drawn by plotting concentration of glucose in blood versus time intervals.

### Estimation of glycogen content in tissue[11]

Collection of tissue: Rats were sacrificed by an overdose of anesthetic ether. The liver and skeletal muscle were immediately excised and chilled in ice cold 0.9% sodium chloride. The tissues were kept at −80°C until processed.

The assay was based on the color reaction, which occurs when a dilute solution of glucose was heated with concentrated sulphuric acid. Since glycogen hydrolyses to give glucose in sulphuric acid, the above principle was used to determine glycogen. The intensity of pink color produced was measured spectrophotometrically at 520 nm.

### Statistical analysis

Data were expressed as mean ± SEM. Comparison between different groups was done using One-way ANOVA followed by Dunnett's multiple comparison test. *P*<0.05 was considered to be statistically significant.

## Results

### Acute toxicity

LD_50_ of the aqueous extract of *Trichosanthes cucumerina* was found to be 1.12 g/kg body weight in mice.

### Effect on fasting blood glucose

Aqueous extract of *Trichosanthes cucumerina* at a dose of 100 mg/kg/day treated orally for six weeks exhibited a significant (*P*<0.01) decrease in fasting blood glucose of NIDDM animals as compared to NIDDM control. Blood glucose of diabetic animals starts decreasing from the 1^st^ week of drug treatment. Further 2 weeks and 4 weeks of drug treatment, decreased the blood glucose of diabetic animals continuously [Table 1]. Difference between 4^th^ week and 6^th^ week of drug treatment is statistically non-significant. Glibenclamide at a dose of 1.5 mg/kg used as standard drug for comparison showed significant (*P*<0.01) decrease in fasting blood glucose of NIDDM animals as compared to NIDDM control.

**Table 1:** Effect of aqueous extract of *Trichosanthes cucumerina* on fating blood glucose of NIDDM induced rats measured after 1, 2, 4 and 6 weeks of drug treatment

*Group*	*Blood glucose (mg/dl)*				
	
	*Before treatment*	*1 week*	*2 weeks*	*4 weeks*	*6 weeks*
Normal	94.33 ± 1.47	94.81 ± 1.61	94.86 ± 1.51	95.40 ± 1.73	95.60 ± 1.74
NIDDM control	182.81 ± 2.85	184.91 ± 3.10	185.66 ± 2.93	188.15 ± 3.0	192.81 ± 3.12
NIDDM treated with TC	183.30 ± 3.19	165.55 ± 2.92**	127.56 ± 2.15**	113.45 ± 2.27**	112.8 ± 2.26**
NIDDM treated with GL	178.52 ± 3.56	97.83 ± 1.55**	103.81 ± 1.61**	106.28 ± 1.98**	107.5 ± 2.51**
One-way-ANOVA F	327.67	368.98	367.05	336.77	355.50
df	3, 20	3, 20	3, 20	3, 20	3, 20

Values are mean ± SEM; n=6;

** *P*<0.01 as compared to NIDDM control (One-way ANOVA followed by Dunnett multiple comparison test); TC: *Trichosanthes cucumerina*; GL: Glibenclamide; NIDDM: Non Insulin Dependent Diabetes Mellitus

### Body weight

**Figure 1:**
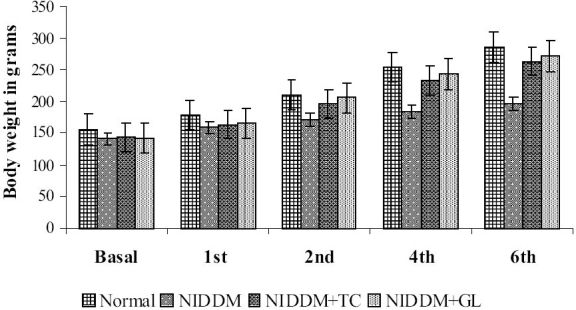
Body weight of Normal, NIDDM control, NIDDM treated with *Trichosanthes cucumerina* (NIDDM+TC) and NIDDM treated with Glibenclamide (NIDDM+GL) groups measured at Basal level (before drug treatment) and after 1st, 2nd, 4th and 6 weeks of drug treatment; n=6; *P<0.05, **P<0.01 compared to NIDDM control; F=208.03 (6thweek); df=3, 20 (One-way ANOVA followed by Dunnett multiple comparison test)

Body weight of streptozotocin induced NIDDM rats were found to be statistically less (*P*<0.01) compared to normal rats at basal level (before drug treatment). After one week of drug treatment, aqueous extract of *Trichosanthes cucumerina* did not improve the body weight of NIDDM rats (*P*>0.05) compared to NIDDM control. Two weeks of drug treatment has significantly (*P*<0.01) increased the body weight of NIDDM animals compared to NIDDM control. Progress in weight gain of animals in drug treated group was observed up to six weeks. Body weight of different groups of animals at basal level and at the end of 1^st^, 2^nd^, 4^th^ and 6 weeks of drug treatment are represented graphically [Figure 1].

### Effect on oral glucose tolerance

**Figure 2:**
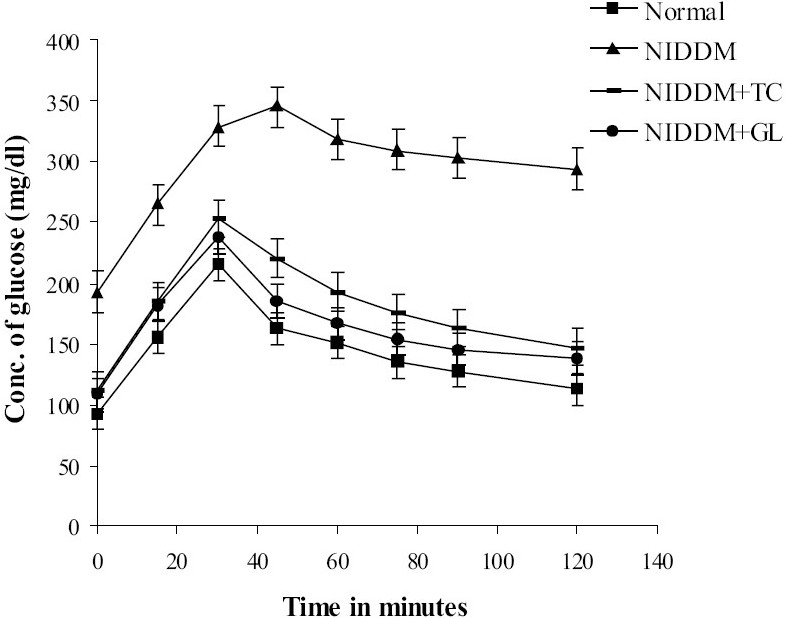
Oral Glucose Tolerance Test curves of Normal, NIDDM control, NIDDM treated with Trichosanthes cucumerina (NIDDM+TC) and NIDDM treated with Glibenclamide (NIDDM+GL) groups; n=6; **P<0.01 compared to NIDDM control; F=851.98; df=3, 20 (One-way ANOVA followed by Dunnett multiple comparison test)

Oral Glucose Tolerance Test (OGTT) of NIDDM animals showed glucose intolerance. In case of normal group, the blood glucose attained peak level at 30 min followed by subsequent fall up to 120 min. In case of NIDDM group, the blood glucose reached peak level at 45 min and remains high even after 2h. In case of the drug treated group, the blood glucose reached peak level at 30 min and subsequently decreased up to 2 h [Figure 2]. Aqueous extract of *Trichosanthes cucumerina* has improved the oral glucose tolerance in NIDDM animals and significantly (*P*<0.01) decreased the elevated postprandial blood glucose of NIDDM animals compared to NIDDM control. Standard drug of comparison, glibenclamide significantly (*P*<0.01) decreased the postprandial blood glucose of NIDDM animals as compared to NIDDM control.

### Effect on glycogen content in liver and skeletal muscle

**Figure 3:**
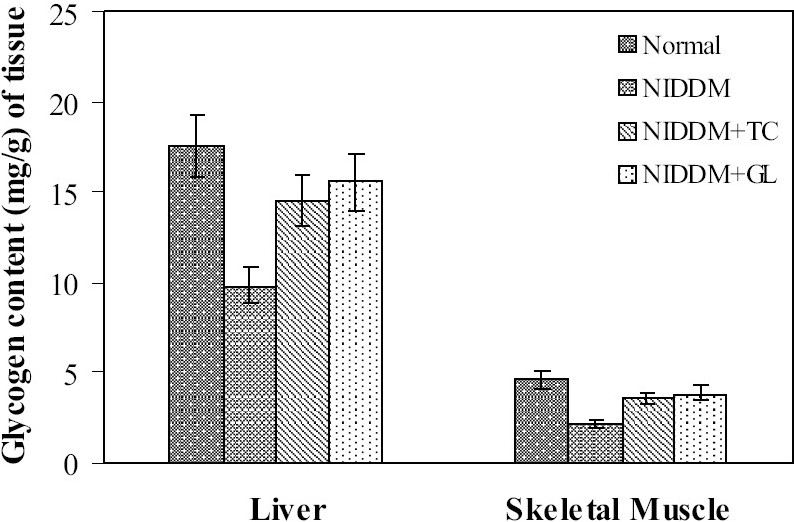
Effect of *Trichosanthes cucumerina* on glycogen content of liver and skeletal muscle. Values are mean ± SEM; n=6; **P<0.01 as compared to NIDDM control; Liver: F=85.63, df= 3, 20; Skeletal Muscle: F=24.62, df=3, 20 (One-way ANOVA followed by Dunnett multiple comparison test); TC: *Trichosanthes cucumerina*; GL: Glibenclamide

Significant (*P*<0.01) increase in glycogen content of liver and skeletal muscle was observed in case of both *Trichosanthes cucumerina* and glibenclamide treated groups as compared to NIDDM control [Figure 3]. Liver and skeletal muscle glycogen content was increased by 62% and 58.8% respectively by the aqueous extract of *Trichosanthes cucumerina*, whereas glibenclamide has increased the same by 76.3% and 70.3% as compared to NIDDM control.

## Discussion

In NIDDM, partial or total deficiency of insulin causes derangement in carbohydrate metabolism.[1] Aqueous extract of *Trichosanthes cucumerina* decreased the fasting blood glucose of NIDDM animals. Decrease in blood glucose started after one week of drug treatment and continued up to four weeks. After four weeks, blood glucose of diabetic animals was found to be maintained within the normal levels as there was no significant difference between four weeks and six weeks of drug treatment. Decrease in blood glucose may be due to cucurbitacin glycosides present in the drug. Cucurbitacin glycosides increase the insulin secretion of pancreatic β-cells.[12] Streptozotocin induced diabetic rats gained significantly less weight compared to normal as well as drug treated groups. The failure of diabetic animals to gain weight during the course of time is due to continuous excretion of glucose in diabetic subjects. Insulin deficiency and insulin resistance of NIDDM decrease the peripheral uptake of glucose and glycogen synthesis thereby affect the body weight of animals. In NIDDM, the kinetics of insulin release in response to meal or glucose alters. Therefore elevated postprandial blood glucose is observed.[13] Postprandial blood glucose may be elevated even in presence of normal fasting blood glucose, constituting an early stage in diabetes.[14] *Trichosanthes cucumerina* has improved glucose tolerance in NIDDM animals. The effect may be due to stimulation of insulin release by pancreas or due to delayed glucose absorption.[15] Delayed glucose absorption is usually due to inhibition of enzyme α-glucosidase located at intestinal surface.[16] Liver plays an important role in buffering the postprandial hyperglycemia and is involved in the synthesis of glycogen.[2,17] In NIDDM, normal capacity of liver to synthesize glycogen is impaired. The activation of key enzymes involved in glycogen synthesis appears to be defective in NIDDM animals. The defect is due to insulin deficiency.[17] Hence glycogen content of insulin dependent tissues such as liver and skeletal muscle in NIDDM animals has markedly decreased. Increase in glycogen content of liver and skeletal muscle by *Trichosanthes cucumerina* indicates the possible drug effect on sensitizing these tissues for uptake of glucose and also enhancing the activity of key enzymes of glycogen synthesis.[18] Drug may improve the insulin mediated upregulation of glucose transporters (GLUT-4) which facilitates the uptake of glucose by the skeletal muscle and thereby increase the glycogen content.[19] Improvement in tissue glycogen content by glibenclamide is mediated through insulin action. The extract may possess insulin like activity.[20] Oral administration of aqueous extract of *Trichosanthes cucumerina* at a low dose (100 mg/kg/day) was found to be effective in the present study. Earlier studies reported the effect of ethanolic extract of *Trichosanthes cucumerina* at a high dose (250 mg/kg/day) for hypoglycemic activity.[4] These observations suggest that the active principle (s) for antidiabetic activity may be of more water soluble nature.

## Conclusion

Studies revealed that the aqueous extract of *Trichosanthes cucumerina* possess potent antidiabetic activity. Drug improved the oral glucose tolerance of NIDDM subjects. Improved tissue glycogen content indicates the effect of drug on uptake of glucose by the peripheral tissues and thereby decreases the insulin resistance of NIDDM. A further study on functional activity of pancreatic β-cells is required to know the drug effect on pancreas. A study on enzymes involved in carbohydrate metabolism and chemical characterization of aqueous extract are necessary to elucidate the mechanism of action.
